# Specifics of Data Collection and Data Processing during Formation of RailVista Dataset for Machine Learning- and Deep Learning-Based Applications

**DOI:** 10.3390/s24165239

**Published:** 2024-08-13

**Authors:** Gulsipat Abisheva, Nikolaj Goranin, Bibigul Razakhova, Tolegen Aidynov, Dina Satybaldina

**Affiliations:** 1 Department of Artificial Intelligence Technology, Faculty of Information Technologies, L.N. Gumilyov Eurasian National University, Astana KZ-010000, Kazakhstan; 950421401010@enu.kz (G.A.); razakhova_bsh@enu.kz (B.R.); 2 Department of Information Systems, Faculty of Fundamental Sciences, Vilnius Gediminas Technical University, LT-08412 Vilnius, Lithuania; nikolaj.goranin@vilniustech.lt; 3 Department of Information Security, Faculty of Information Technologies, L.N. Gumilyov Eurasian National University, Astana KZ-010000, Kazakhstan; 951005351309@enu.kz

**Keywords:** dataset, data collection, machine learning, railway, railway track defects

## Abstract

This paper presents the methodology and outcomes of creating the Rail Vista dataset, designed for detecting defects on railway tracks using machine and deep learning techniques. The dataset comprises 200,000 high-resolution images categorized into 19 distinct classes covering various railway infrastructure defects. The data collection involved a meticulous process including complex image capture methods, distortion techniques for data enrichment, and secure storage in a data warehouse using efficient binary file formats. This structured dataset facilitates effective training of machine/deep learning models, enhancing automated defect detection systems in railway safety and maintenance applications. The study underscores the critical role of high-quality datasets in advancing machine learning applications within the railway domain, highlighting future prospects for improving safety and reliability through automated recognition technologies.

## 1. Introduction

In modern transport construction, railways play a key role in ensuring the safety and efficiency of movement. In recent years, rail transport has begun to expand globally and acts as the most preferred form of transportation worldwide [1].

The design of a railway track consists of rails and sleepers, fastening elements, and ballasts for the movement of trains and vehicles.

The safety of train traffic directly depends on the condition of the railway tracks. Therefore, early detection of defects in railway transport is important [2]. Until now, contact measurement methods with human participation have been used to identify faults on rails.

Recent advancements in railway infrastructure inspection have witnessed a significant shift towards automated methods, reducing reliance on human-operated contact measurement techniques. Reference [3] pioneers the integration of unmanned aerial vehicles (UAVs) with cutting-edge machine vision technologies. By employing sophisticated image processing algorithms and machine learning, this approach enhances inspection precision, accelerates the assessment process, and mitigates the costs and risks associated with manual inspections.

Reference [4] contributes to this burgeoning field by focusing on the development of autonomous inspection systems. Leveraging vision-based technologies, this research underscores the real-time defect detection capabilities of autonomous platforms, which significantly diminish the necessity for human intervention in inspection tasks. Collectively, these studies underscore the transformative impact of intelligent machine vision and autonomous systems in revolutionizing railway infrastructure inspection practices, heralding a new era of enhanced safety and operational effectiveness.

With the increase in the use of railways, the need for enhanced control over transportation safety increases [5,6]. Railroad tracks are subject to various types of defects that can have a significant impact on the safety and efficiency of train operations. Typical defects include cracks in the rails, wear on the rail surface, deformations, fistulas, and defects in welded joints. They can occur due to overuse, exposure to weather conditions, insufficient maintenance, or wear and tear of materials. These defects require systematic monitoring and prompt elimination to ensure the safety and reliability of railway traffic.

Therefore, the purpose of this article is to describe the methods of creating, and significance of, a dataset for detecting defects on railway tracks using machine and deep learning methods. The main focus is on the creation and curation of the “RailVista” dataset, which is an extensive and organized set of images with markup necessary for machine learning and deep learning models.

The article describes a detailed data collection process that includes the process of taking images of railway tracks, using binary files to store images in a data warehouse for quick access and optimal use of disk space.

An important goal of the work is to develop an annotated dataset covering a wide range of railway track defects. This dataset will facilitate the development and evaluation of machine learning algorithms aimed at automating defect detection and classification.

To improve the safety of railway transport, speed up the track inspection process, reduce the time and financial costs of maintenance, and reduce the labor costs of workers, the use of advanced technologies is proposed [7]. The transition from traditional human railway inspection to machine learning techniques represents a significant step forward in ensuring transportation safety and efficiency.

Modern advances in machine learning have significantly contributed to improving the effectiveness of various computer vision tasks. Technologies are also actively used in the railway sector [8]. Deutsche Bahn uses machine learning to optimize train schedules. In 2022, the use of artificial intelligence has reduced train delays by a total of 58 thousand minutes [9]. General Electric has successfully applied machine learning to create smart locomotives and reduce disruptions in the rail transportation system. As part of a joint project with Deutsche Bahn, they managed to achieve a record level of train efficiency, increasing it by 25% [10].

Notable breakthroughs have been noted in the field of image classification [11] and object detection [12,13]. Detection and recognition of railway tracks based on image processing methods has been an active area of research in the last decade [14].

Our work is designed to emphasize the need for high-quality datasets and the importance of their detailing to improve the accuracy and reliability of defect detection systems, contributing to the further development and improvement of transport security technologies in railway transport.

The study underscores the critical role of high-quality datasets in advancing machine learning applications within the railway domain. By addressing the scarcity of publicly available, annotated datasets specific to railway track defects, Rail Vista fills a significant gap in the field. The dataset’s comprehensiveness across 19 defect categories allows for robust model training, promoting accurate and efficient automated defect detection. This capability is pivotal for ensuring railway safety and reliability, minimizing operational disruptions, and reducing maintenance costs.

In conclusion, this research contributes not only by providing a large-scale, meticulously curated dataset but also by demonstrating the transformative potential of machine learning in enhancing railway infrastructure maintenance and safety. The outcomes highlight future prospects for advancing automated recognition technologies in the railway sector, paving the way for more proactive and efficient maintenance practices and ultimately improving overall railway system reliability and safety standards.

The results of this research encompass the following:

Data Collection and Aggregation: Successful creation of an extensive dataset comprising over 200,000 records about various types of defects on railway tracks, including cracks, wear, rail bulges, and misalignments.

Detailed Metadata Creation: Development of a metadata system containing information about geolocation, data collection timing, environmental conditions, and additional parameters that facilitate a more profound analysis of defects.

High-Resolution Images: The inclusion of high-quality images in the dataset enables meticulous examination of defects, forming a foundation for the development of computer vision algorithms.

Diversity of Defects: The dataset encompasses diverse scenarios and types of defects, rendering it a valuable resource for training and validating machine learning models and defect-detection algorithms.

Practical Application in Automation: The potential utilization of this dataset for developing and testing machine learning algorithms aimed at automated defect detection on railway tracks, to enhance railway transportation safety and efficiency.

This article is devoted to creating a dataset, combining data collection information, data structure, as well as classes of this data. The dataset contains data with detailed descriptions. It also contains information about data collection error information and recommendations for data collection.

The scientific article structure is as follows: The Introduction sets the stage by addressing the significance of detecting railway track defects, outlining research goals, and summarizing existing methods. Section 2 analyzes prior research on detecting defects, reviewing methods used and evaluating their effectiveness. Section 3 describes dataset creation methodologies, data collection processes, metadata creation, image generation, and data processing. Section 3.5. details dataset characteristics, record types, storage details, and organization. Section 4 discusses the importance of a high-quality and extensive dataset for the successful detection of defects on railway tracks. Also, an example of the practical application of a dataset is demonstrated in real engineering conditions. Section 5 summarizes key findings, emphasizes practical significance, and proposes future research avenues.

## 2. Prior and Related Work

In recent years, machine learning has revolutionized various fields such as computer vision, natural language processing, and speech recognition. With the increasing volume of data collected by monitoring devices, such as wireless sensor networks or high-resolution video cameras, widely used to monitor critical railway infrastructure, machine learning is becoming an increasingly popular means to improve the efficiency and reliability of railway transport systems [15].

The dataset encompasses a spectrum of commonly observed defects inherent to railway track infrastructure. These defects comprise structural anomalies such as fissures, fatigue-induced wear, rail distortions, and deviations in track alignment. Each defect category exhibits distinct characteristics, varying degrees of severity, and diverse spatial distributions across the track network, presenting challenges for accurate identification and remediation.

Human-based inspection methodologies face inherent limitations within railway track monitoring. Human inspectors are subject to limitations in sustained concentration, leading to the potential oversight of subtle defects or irregularities. Moreover, variability in inspector expertise and subjectivity can affect the consistency and reliability of defect identification. Environmental factors, including adverse weather conditions or restricted access to certain track segments, pose additional challenges, impeding comprehensive and uniform inspection coverage across the railway network.

Methods based on computer vision can identify and identify defects based on the data obtained. In the work [16,17,18,19] of the authors, computer vision is used to collect data, and a high-resolution camera is placed under the measuring train, taking images of the rails during the movement of the train. The resulting images are analyzed using computer image processing techniques. Problems related to various rail components are classified as cracks, corrugations, bolt defects, fastening defects, and rail surface defects.

Reference [20] discusses two approaches to improving the accuracy of machine learning: creating more sophisticated models and using higher-quality datasets. The authors argue that more research is needed on how dataset quality impacts models. The article proposes a new method for validating datasets alongside a machine learning system. This method is based on metamorphic testing, which involves creating tests that adequately assess the system. The effectiveness of the proposed approach is demonstrated through the example of automatic classification of images of biological cells.

Reference [21] focuses on improving the detection of fastener defects in railway tracks using deep learning techniques. The authors propose an intelligent algorithm named YOLO-Fastener, which is designed to efficiently and accurately identify defects in ballastless track systems. The study highlights the advantages of this approach, including enhanced detection accuracy and the potential for real-time application in ensuring the safety and reliability of high-speed rail operations.

Reference [22] presents a deep learning approach to detect cracks in slab tracks at the pixel level. The study introduces a method that combines multi-scale feature fusion to enhance the detection accuracy. This technique allows the model to capture detailed features at different scales, leading to more precise and reliable identification of cracks. The effectiveness of this approach is validated through extensive experiments on real-world slab track images, demonstrating significant improvements in crack detection performance.

In [23], the dataset provides information for calculating the residual fatigue life of concrete sleepers in railway systems based on data collected in real operating conditions. The corresponding article published by the authors describes in detail the methodology used in data collection to predict the durability of an asset [24]. This dataset can be effectively used to create predictive maintenance models and monitor the state of the system. Additionally, the dataset in [25], also collected by ProRail using a similar methodology, contains images of insulation seams in the railway network. The third dataset from ProRail [26] includes images of insulation seams and color masks designed to detect spark erosion on railway tracks. These datasets provide material for training artificial intelligence models, such as convolutional neural networks (CNN), to identify and localize railway assets, as well as predict possible deviations.

The dataset in [27] contains signals characterizing the deviation of the railway track and obtained by measuring speed and acceleration. Including both simulated and measured train movement data, this dataset is designed to classify total deviation and downward deviation based on train movement records. It contains data on the state of sleeping and well-sleeping rails, as well as a model of a well-sleeping rail. The records were obtained using inertial sensors, while the simulated data were generated using the corresponding equations.

In [28], a systematic review of datasets for railway applications was conducted. A small fraction of these datasets provide detailed documentation, and only a few have published articles containing comprehensive descriptions. Most datasets lack information regarding data collection methodologies. Information about potential errors, recommendations, or other limitations is often absent. The deficiency in documentation may impede the assessment of data transparency and reliability.

Furthermore, the absence of suitable image datasets in the railway transportation domain is noted. Very few datasets reviewed are related to images and available for widespread use. This significant limitation is especially noteworthy considering the broad spectrum of research utilizing computer vision models in this field.

Additionally, a notable issue identified is the lack of metadata for the available datasets. Most of these datasets lack comprehensive information regarding data collection methodologies, hindering the verification of data transparency and accuracy. Inadequate information about data collection methods, the absence of data labeling mentions or limitations, and the scarcity of publications with detailed descriptions of the datasets pose obstacles to identifying and analyzing this field and utilizing this information in scientific research.

We conducted a comparison of several open datasets related to railway track defects (Table 1). During the analysis, it was found that many of these datasets lack detailed information about the data collection methods. This means that there is no clear description of how the data on defects were collected and classified.

Furthermore, most of them also lack pre-existing classification of data regarding defects. Such lack of structure complicates the analysis and utilization of the data for developing defect-detection algorithms and improving railway track safety.

Railway track defect detection is crucial for ensuring the safety and reliability of rail networks. Traditional manual inspections, while effective, are labor-intensive and prone to human error. To enhance accuracy and efficiency, various automated methods have been developed. These include ultrasonic testing, which uses high-frequency sound waves to detect internal flaws; magnetic flux leakage, which identifies surface and near-surface defects by measuring changes in magnetic fields; and vision-based systems, which utilize cameras and image processing algorithms to detect visual anomalies. Advanced techniques like LiDAR (Light Detection and Ranging) and machine learning are also being increasingly integrated to improve detection capabilities and data analysis. These modern methods provide more comprehensive and reliable assessments, ensuring timely maintenance and reducing the risk of accidents and service disruptions.

There are technologies that allow you to detect damage or excessive displacement of noise-canceling elements relative to the rail (rail shock absorbers), which further increases safety and operational reliability [29]. This opportunity highlights the importance of introducing advanced control techniques into railway maintenance practices.

In addition to advancements in detection technologies like ultrasonic testing, magnetic flux leakage, and vision-based systems, ongoing research explores innovative materials for railway components. For instance, recent studies [30] investigate the use of recycled rubber granules from tears as an elastic coating for prototype rail dampers, focusing on enhancing their operational durability. Such initiatives not only aim to improve the performance of rail infrastructure but also contribute to sustainable practices by utilizing recycled materials effectively.

Table 2 provides a comparative overview of methods applied in railway track defect detection. It summarizes key approaches and obtained results, and identifies research gaps from various studies.

Moreover, in recent years, the role of deep neural networks in fault detection in predictive modeling in industrial environments has also been developing. A study of GRU (Gated Recurrent Unit) forecasting methods for the industrial sector has shown good results [34]. These methods use various preprocessing techniques to refine forecasts, thereby increasing the efficiency and accuracy of production processes. Such innovations are an example of continuous efforts to introduce advanced technologies into industrial activities, providing not only increased reliability, but also optimized productivity in various industries.

## 3. Materials and Methods

This section encompasses a detailed explanation of the methodologies utilized for data collection in the railway track defect detection dataset. It covers the specifics of the data collection method, the format used for storing photographic files, and the process involved in data markup.

### 3.1. The Equipment Used and Project Framework

The choice of camera for capturing railroad defects depends on several factors, including:

- Resolution: A camera with good resolution will provide detailed images of defects.
- Optics: High-quality optics provide clear images with a high level of detail.
- Shooting speed: Some defects may appear when moving, so a camera with a high shooting speed may be preferable.
- Shooting Conditions: If you are shooting in low-light conditions or varying climates, you need to choose a camera that can handle those conditions well.

**Figure 1:**
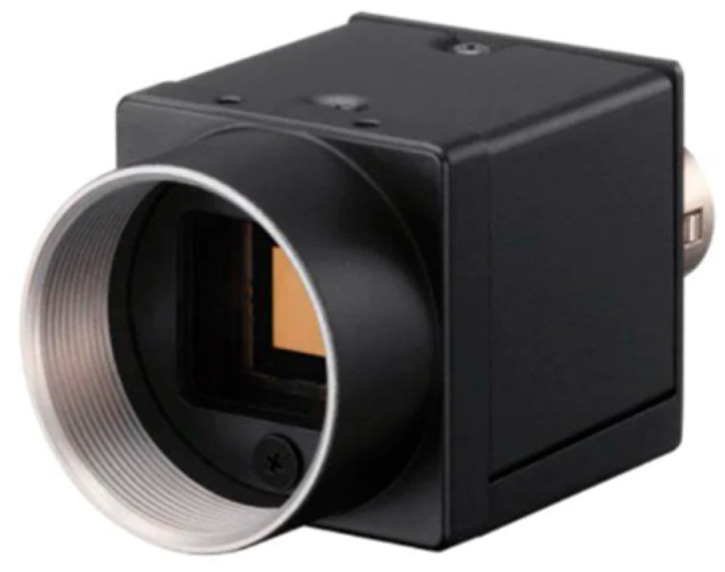
Camera Sony XCG-CG160.

Certain camera models from different manufacturers may be suitable for filming railroad defects. Cameras from Sony, Canon, Nikon, and other manufacturers can offer models with high resolution, high-quality optics, and the ability to shoot under various conditions. To collect railway track data, data obtained using cameras of the SONY XCG-CG160 model, manufactured by Sony Corporation in Tokyo, Japan, were used, as shown in Figure 1.

Table 3 compares the Sony XCG-CG160 camera with other models commonly used for capturing defects on railway tracks. The table includes key characteristics such as resolution, pixel size, interface, frame rate, dynamic range, and weight. This comparison can assist in making an informed decision when selecting a camera for railway track defect detection applications.

The Sony XCG-CG160 model stands out with its high image resolution of 1920 × 1200 pixels and a pixel size of 3.45 µm, which can provide sharper and more detailed images of defects on railway tracks. Additionally, it boasts a moderate capture speed of 35 frames per second. The XCG-CG160 incorporates the latest Pregius GSCMOS (Global Shutter CMOS) sensor, providing an optimal combination of resolution and frame rate for use in production environments and inspection applications, where GigE architecture is preferred for data transmission. This series offers significant technological improvements in sensitivity, dynamic range, noise reduction, and frame rate capabilities.

The XCG-CG160 features a resolution of 1456 (horizontal) × 1088 (vertical) effective pixels, delivering a frame rate of 70 frames per second. It supports the GigE 1.2/2.0 interface and enables data transmission through a single cable with Power over Ethernet (PoE) capability [35].

Sony cameras specifically designed for detecting defects on railway tracks are engineered considering the intricate conditions typical of traffic management systems. Their components are carefully selected to ensure reliable operation in various weather conditions, including adverse weather, strong winds, and temperature fluctuations, allowing for efficient functioning within railway infrastructure settings.

Image optimization in Sony cameras is not limited to the selection of shutter speed and gain. All aspects of processing, from noise filtering and gain control to autofocus and day-night mode switching, have been completely rethought and tested in practice in various moving scenarios.

#### 3.1.1. IR Illumination

Shooting at night requires the use of artificial lighting. Typically, infrared (IR) lighting is used in such cases; it is invisible to the eye and does not cause glare. IR light significantly improves the visibility and contrast of objects in dark conditions or when visibility is poor due to cloudy weather. This infrared illumination can be created by LEDs built into the camera or external light sources placed relative to the camera.

#### 3.1.2. Camera Resolution

The camera resolution is the standard Full HD shooting format (1920 × 1080). The distance from the camera to the object is 1–2 m. If the distance is greater, then the camera resolution needs to be higher. To get a good result, you need to set the camera directions to 90°.

It is also extremely important that the images in the data are as close as possible to the real conditions in which the neural network model will function. Before starting data collection, it is necessary to determine exactly which images the model will accept as input, where the camera is located, and what resolution it has. For example, if the camera has a low resolution and captures small images, then the photos in the dataset should also be small. Also take into account the working conditions of the camera; for example, if it is installed in a room with high humidity, the images for training may be slightly blurred or contain water droplets for more effective training.

#### 3.1.3. The Framework of This Project

The project to create a dataset of railway track defects has the following structure:

1. The purpose of the project: To create a dataset containing a variety of railway track defects for subsequent training and evaluation of models.
2. Project Components:
  – Data collection: The use of geodetic instruments and high-resolution cameras to collect a variety of data on the condition of rails and nearby infrastructure.
  – Data processing: The use of MATLAB R2021b (version 9.11), Python 3.9 with NumPy 1.21.5, Pandas 2.2 libraries for processing, filtering, and analyzing data received from sensors and cameras.
  – Data markup: Using annotation tools, marking up images containing defects on railway tracks.

### 3.2. Data Collection Method

**Figure 2:**
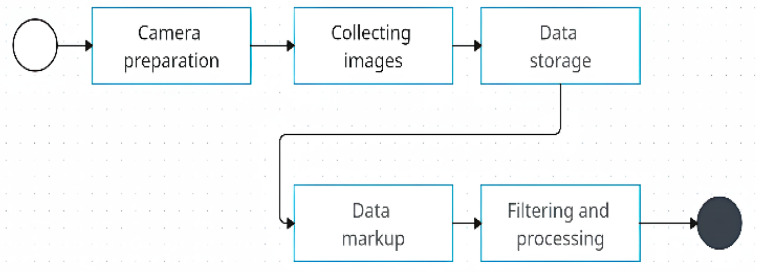
Method of data collection.

The data collection method and processing for detecting defects on railway tracks are shown in Figure 2. The method includes several key steps, each of which is aimed at maximizing the accuracy and efficiency of the data.

1. Preparation of the camera: The placement of specialized Sony cameras in the control area is carried out. Camera settings are optimized.
  - When configuring, pay attention to the following features:
  - Set the data collection frequency to ensure seamless and minimally repetitive data gathering with the current window sizes adjusted.
  - Allocate a dedicated processor core for both data reception and processing for each camera. Do not assign missing processor cores, as this may cause the program collecting data to freeze.
  - For each camera, allocate a separate hard drive for storing “raw data”. You are allowed to use one Solid State Drive (SSD) to store data from two cameras.
2. Collecting images: Images are collected automatically using Sony cameras according to the request.
  - A complete and more detailed description of automatic image collection using cameras is presented in Section 3.4 of this article.
3. Data storage: The resulting images are stored in a database with a suitable structure.
4. Data markup: Defects in images are marked manually by specialists.
5. Filtering and processing: Data are filtered to remove unnecessary information. Images are processed to prepare them for model training.

In the course of collecting data for the analysis of railway track defects using a Sony camera, we encountered the problem of image deformation at the edges. This deformation can be caused by various factors, such as lens distortion, less-than-ideal lighting conditions, or other atmospheric influences.

To improve the quality of the input data before training the deep learning model, we used a procedure for cropping images at the edges (crop). This data processing step aimed to remove distorted areas, focusing the model’s attention solely on the central part of the image, where the deformation was minimal.

**Figure 3:**
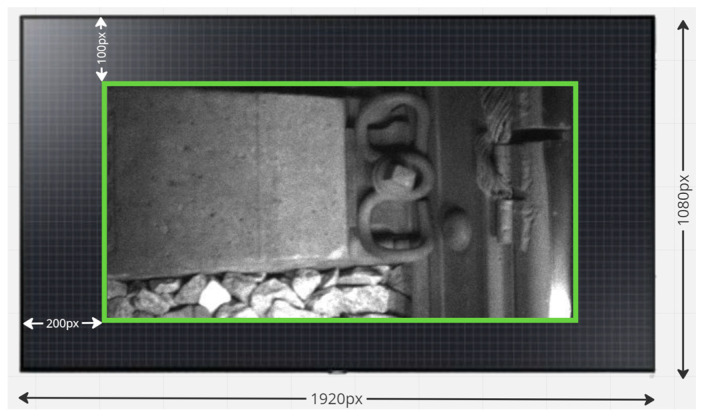
Example of cropping an image at the edges.

Cropping images at the edges improves the quality of model training because it is trained on clearer, more representative data that are free of distortions. An example of image cropping is shown in Figure 3. In addition, this process also helps reduce the influence of artifacts caused by edge deformation on the model results.

Thus, the use of image cropping becomes an important step in data preprocessing, providing more accurate and efficient model training for railway track defect detection.

### 3.3. Photo File Storage Format

The images collected are stored in binary files. Storing images in binary files provides several advantages, especially when working with a large amount of data. Table 4 below presents a comparison of image storage formats.

Image file names were created according to the following rules: YYYY_MM_DD_MM_SS.sH, where YYYY_MM_DD_MM_SS is the date and time the file was created, s is the file with photos. The first 8 bytes of the file are the header of two fields, which is presented in Table 5.

After the title, photos are written in the form of a two-dimensional array with the dimensions specified in the file header. The first line contains service information as in Table 6.

### 3.4. Data Markup Process

#### 3.4.1. Expert Involvement in Data Markup

Experts in the field of railway transport actively participated in the process of marking up the data for our study.

The images were marked up manually, with the recommendations of experts, and their experience and knowledge of the subject area played a key role in creating accurate and reliable annotations. This approach provides a high standard of markup, which is critical for the subsequent training of machine learning models. Each image was marked by only one expert.

In the process of classifying defects on railway tracks, we actively used instructions. This document has been a key resource for defining the standards and criteria applicable to railway infrastructure, including the classification of defects and their characteristics.

#### 3.4.2. Optimization of Data Collection through Correlation

Correlation in image processing was used to optimize data collection. In image processing, measuring the degree of similarity between different areas of images or between an image and its processed version plays a key role. One of the effective methods for this task is the use of correlation, especially cross-correlation.

1. Cross-correlation in image processing.

Cross-correlation is a statistical measure used to assess the degree of relationship or similarity between two variables. In the context of image processing, cross-correlation is often used to measure similarity between two areas of an image or between an image and its processed variant, such as a template.

2. The formula for discrete cross-correlation.

The equation for the discrete cross-correlation of two signals f and g of length N is as follows:

(1) $$\left(f*g\right)\left[n\right]=\Sigma_{m=0}^{N-1}f\left[m\right]*g\left[m+n\right]$$

where *n*—is the shift and (*f* ∗ *g*) [*n*] is the cross-correlation value for the shift of *n*.

Application of the cross-correlation method:

cv2.matchTemplate (image, template, cv2.TM_CCOEFF_NORMED).

In the context of image processing, cross-correlation is often used when searching for patterns. The process can be represented as follows:

1. Selection of the template and the main image.
2. Calculation of cross-correlation between the template and various areas of the main image.
3. Finding the position with the maximum cross-correlation value.

If the cross-correlation value is high in a certain area, it indicates a strong match between the template and this area of the main image.

The code that was provided earlier used normalized cross-correlation (cv2.TM_CCOEFF_NORMED), which scales the cross-correlation values to an interval from −1 to 1. This is useful for more convenient threshold determination and interpretation of the results.

#### 3.4.3. Specialized Program for Image Markup

**Figure 4:**
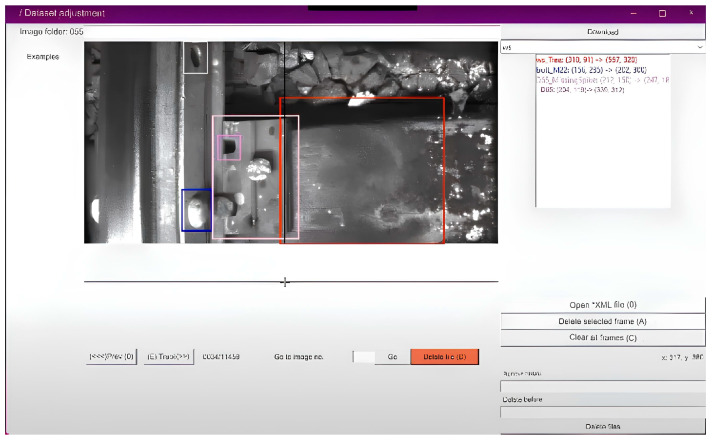
Image markup program.

In the process of marking defects in images, a specialized program for marking data was used. This program provides a user-friendly interface for experts, allowing them to identify and classify defects, as well as assign appropriate coordinates. The program facilitates the markup process, making it more efficient and accurate. An example of the application is shown below in Figure 4.

Using such a program speeds up the annotation process and reduces the likelihood of errors, which is important to ensure the receipt of high-quality data. Experts in the field of railway track defects can easily interact with the program by highlighting and classifying defects in images.

### 3.5. The Dataset Structure

In this section, we will present the structure of the dataset used for detecting defects on railway tracks. A detailed description of the dataset’s structure will provide a better understanding of the data organization, which is crucial for subsequent analysis and machine learning model training.

#### 3.5.1. Data Structure

The following key components are present in the dataset created:

- Images and dimensions: All images have the same format and dimensions, which ensures uniformity of input data for the model.
- Resolution: High resolution is used for more detailed analysis.
- Tags: Each image in the dataset is provided with appropriate labels that indicate the location and type of identified defects or objects on the railway track.
- Coordinates: Coordinates (xmax, ymax, xmin, ymax) are specified for each selected object, which ensures accurate positioning on the image.
- Object classes: Defects and components of the railway track are classified according to their types.
- There is a clear mapping of each object to a specific class, which facilitates the process.

**Figure 5:**
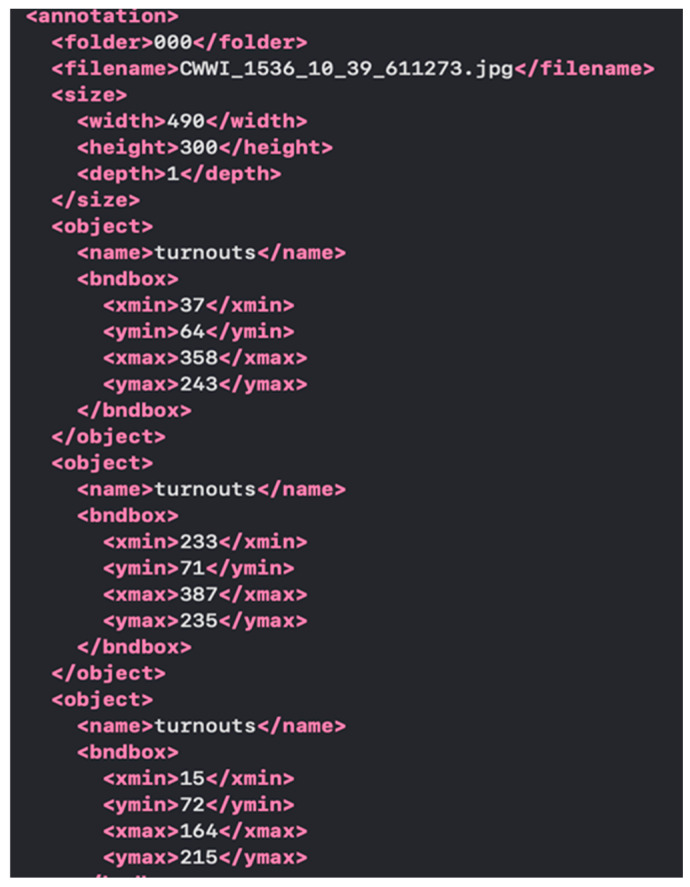
Part of the dataset data structure.

The data structures in XML format for the dataset look like Figure 5.

In this example, each element of the array is a separate image with its file path and corresponding labels. The labels include the class of the object (defect) and the coordinates of its location on the image. A detailed description of the data structure is given below in Table 7.

#### 3.5.2. Truck Dataset Classes

In this section, we present a description of the types of data classes in the dataset, providing detailed characteristics of each class. Table 8 provides the categories of classes presented in our dataset.

Table 8 presents a detailed classification of data derived from the dataset. In the presented table, the analysis of the data objects was carried out with the determination of the presence or absence of defects in the images. The classification covers the key characteristics of the data presented in the dataset, including categories of objects and their corresponding definitions, which provides systematization and a clear understanding of the main parameters of the dataset.

## 4. Discussion

To achieve an effective classification, it is necessary to have an extensive set of samples. In this study, the images of defects were classified by specialists with deep knowledge in this field. In the study, we used 200,000 images. In the pursuit of comprehensive data acquisition, we amassed a vast raw dataset totaling 2 terabytes. Through meticulous curation and annotation, we successfully distilled and labeled 200,000 images, amounting to a refined dataset with a storage footprint of 50 gigabytes. This meticulous process ensured a rich and expansive resource for subsequent analysis and experimentation in our scientific endeavors. A dataset was formed, including 19 classes of marked rail surface defects defined in Table 8, based on real images of railway tracks.

These data not only contribute to improving the overall safety and reliability of railway systems but also provide valuable information for further improvement of methods for detecting and classifying defects. The expansion of the database of defects based on images makes it possible to more accurately train artificial intelligence systems, which in turn contributes to improving the efficiency and accuracy of diagnostics on rails. The results obtained open up new horizons in the field of monitoring and maintenance of railway infrastructure, thereby supporting the evolution in the field of safety and efficiency of railway transport.

An illustration of the application of solutions in the context of the dataset is presented below.

The dataset, which includes images and sensor data with high-frequency measurements, can be used to train machine learning models capable of detecting cracks in rails. In mobile trains equipped with such systems, it is possible to conduct regular checks of the condition of the rails in real time with trained data. This allows you to quickly identify defects and take measures to eliminate them before they lead to serious accidents.

To date, mobile trains equipped with modern crack detection systems are actively using trained data to improve the safety and efficiency of railway infrastructure maintenance.

**Figure 6:**
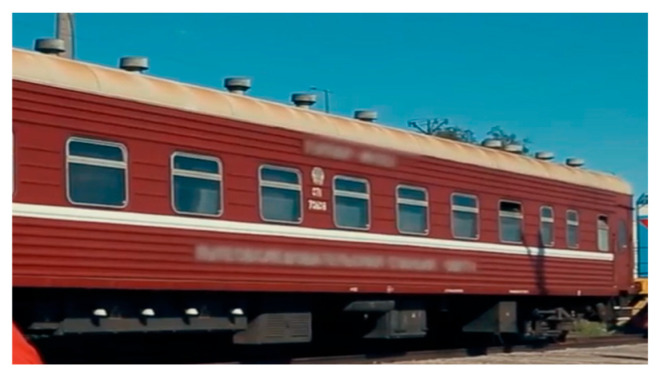
Mobile trains.

**Figure 7:**
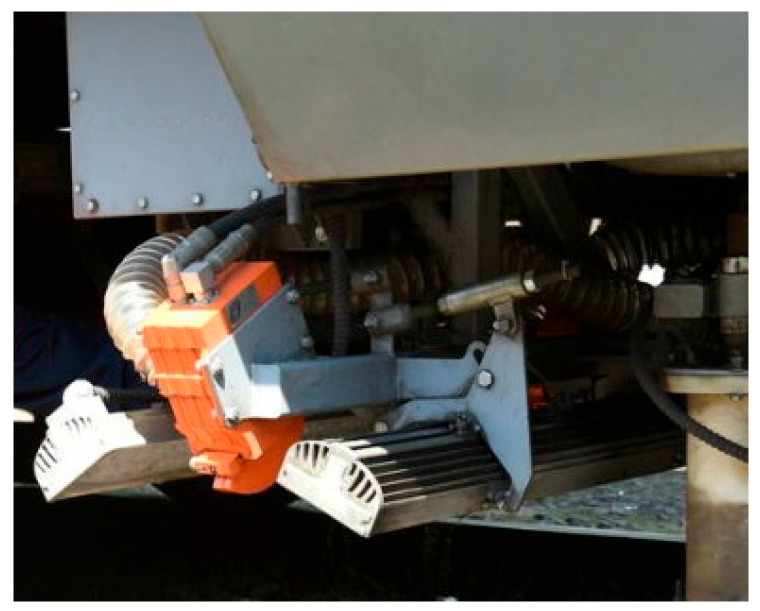
Installed equipment in the train.

In Figure 6 and Figure 7 various sensors and cameras are installed in mobile trains of this type, which collect data in real time. The train has cameras and ultrasonic sensors that scan the rails while moving.

Machine learning algorithms analyze the collected data to identify microcracks, allowing preventive repairs to be carried out before serious damage occurs. If a defect is detected, the system generates a report indicating the exact location and characteristics of the defect, which is sent to the control center for action.

**Figure 8:**
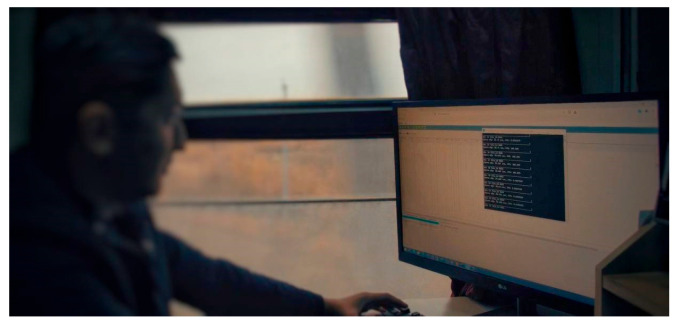
The workplace of the employees in the carriage.

In Figure 8, the image shows the workstation inside the inspection carriage where employees monitor and analyze the incoming data. The system continuously processes the data collected by the sensors and cameras, providing real-time insights into rail conditions. This setup allows for immediate intervention when potential issues are identified, thus significantly enhancing the safety and reliability of railway operations.

These examples demonstrate how the dataset is utilized in real-world engineering applications, emphasizing its critical role in the maintenance and safety of railway infrastructure. The integration of advanced sensor technology, high-resolution imaging, and machine learning algorithms showcases the practical benefits of the dataset in detecting and addressing rail defects efficiently.

In the future, it is planned to train models based on these data, as well as the selection of the best algorithms capable of efficiently processing and analyzing information. These efforts are aimed at further improving the railway infrastructure system using advanced methods and technologies in the field of artificial intelligence and machine learning.

Additionally, as part of our research, we propose to develop a decision-making program that will use a trained model for automatic data analysis. This program will facilitate the rapid identification and classification of defects, which in turn will ensure faster and more efficient maintenance of the railway infrastructure.

Thus, the prospects of our work include not only the development of an advanced model for detecting defects but also the creation of a tool for making operational decisions aimed at ensuring the safety and reliability of railway tracks.

## 5. Conclusions

During the literature review, it was revealed that machine learning is becoming an integral element in the field of defect detection. It provides a high degree of automation and accuracy in identifying defects, surpassing traditional methods. The use of machine learning algorithms is especially relevant in the context of complex and dynamic systems, where hidden or hard-to-reach defects can be easily missed by the human eye.

The proposed method is based on template matching correlation for automating the creation of a dataset for railway infrastructure objects. This approach ensures high efficiency in detecting and classifying railway elements, minimizing manual intervention. The key advantages of the method include high accuracy in object extraction, increased data processing speed, and ease of integration into automated dataset creation systems, making it a valuable tool for researchers and developers in the field of railway technology.

The dataset collected using the proposed method offers a comprehensive and accurately annotated collection of railway infrastructure images. Through the application of template matching correlation, the dataset ensures precise identification and classification of diverse railway elements. This curated dataset not only streamlines research endeavors but also serves as a robust resource for training and validating machine learning models in the field of railway technology, contributing to advancements in automated recognition and analysis of railway objects.

## Figures and Tables

**Table 1:** Comparing datasets on railway track defects.

Dataset	Data Volume	Types of Data	Data Collection Method	Classification of Defects
Rail-5k	5000 images	Rail images	Captured by a trolley moving at 10–20 km/h	6 types (spalls, cracks, joint defects, corrugation, rust, wear)
Vision-Based Inspection of Rail Surface Defects Using Deep Learning	1000 images	Rail images	Captured by a camera mounted on a train	4 types (spalls, cracks, rust, delamination)
Method for Rail Surface Defect Detection Based on Improved Faster R-CNN	2500 images	Rail images	Captured by a camera mounted on a train	5 types (spalls, cracks, joint defects, rust, wear)
Automatic Detection of Rail Surface Defects Using Deep Convolutional Neural Networks	800 images	Rail images	Captured by a camera mounted on a train	3 types (spalls, cracks, rust)
RailVista	200,000 images	Rail images	Captured by a camera mounted on a train	19 types (more details in Table 8)

**Table 2:** Comparative information on methods in railway defect detection.

Reference	Method Used	Main Approach	Obtained Results	Research Gaps
Ji et al. [31]	Deep Learning	Involves the utilization of various deep learning models	Enhanced robustness and accuracy compared to traditional methods	Need for larger, more diverse datasets to further improve model generalization
Han et al. [32]	Machine Learning	Utilizes a modified Faster R-CNN to extract clevises from catenary images	Faster R-CNN architecture outperforms the original and other state-of-the-art object detection models in clevis extraction	The performance of the method with different types of rail infrastructure or with less controlled image capture conditions
Chandran et al. [33]	Deep Learning	Utilizes image processing techniques to enhance fastener position accuracy and remove redundant information from images	Effectively identified various types of rail defects	Problems of practical implementation, such as differences in image quality and environmental conditions, are not considered.

**Table 3:** Comparison of railway track defect detection cameras.

Characteristic	Sony XCG-CG160	Basler acA640-750uc	FLIR Blackfly S BFS-U3-16S2C-CS	IDS UI-5240SE-M-GL
Resolution	1920 × 1200	640 × 480	1920 × 1200	752 × 480
Pixel Size	3.45 µm	7.4 µm	5.86 µm	6 µm
Interface	GigE Vision	USB 3.0	USB 3.1 Gen 1	GigE Vision
Frame Rate	35 fps	82 fps	15 fps	74 fps
Dynamic Range	63 dB	57 dB	60 dB	60 dB
Weight	160 g	220 g	77 g	75 g

**Table 4:** Comparison saving images.

Feature	Jpg Image Saving	Binary Image Saving
Quick read	Usually slower	Faster
Protection from human eyes	Readable and editable	Requires special tools for viewing and editing, provides a higher degree of security
Image quality	The format uses lossy compression, which may result in some loss of image quality. However, it strikes a good balance between quality and file size	Saving in a binary format usually saves data losslessly, which means full quality is preserved, but it can also take up more space
Data integrity	May be subject to editing errors	More resistant to data errors, harder to accidentally change in case hashing is used
File size	Files in this format usually have a smaller size compared to binary formats, which saves storage space	Files in binary format can be large due to saving full information without compression
Complexity of the analysis	The human-readable format facilitates analysis	Requires special tools for analysis and debugging

**Table 5:** 8 Bytes of the file.

Data Type	Offset from the Beginning in Bytes	Appointment	Example
U32	0	Photo width	DO 02 00 00 This number is 720
U32	4	Photo width	FA 00 00 00 This number is 250

**Table 6:** File sizes.

Data Type	Offset from the Beginning in Bytes	Appointment	Example
U64	0	The sign at the beginning of photo 0xF532F532F532F532	Decimal 17,668,453,887,338,804,530
DBL (8 byte)	16	Date and time value in nanoseconds from 1 January 1904	0x43C97B35660B0CAF 3,672,239,970,943,852,030 ns
U32	28	U32CadrCouter The frame number from the camera	Created by the camera itself. Increasing the frame number by more than one indicates a problem with the speed of reading frames from the camera (buffer).

**Table 7:** Description of the data structure.

Element	Meaning	Description
folder	000	The name of the folder where the image is stored
Filename size	CWWI_1544_4_62_579556.jpg	Image file name
width	490	Contains information about the size of the imageImage width in pixels
height	300	Image height in pixels
depth	1	Image depth (1 for monochrome, 3 for color)
name	turnouts	Name of the object class
xmin	28 pixels	Minimum X-axis coordinate (horizontal)
ymin	55 pixels	Minimum Y-axis coordinate (vertical)
xmax	355 pixels	Maximum X-axis coordinate (horizontal)
ymax	202 pixels	Maximum Y-axis coordinate (vertical)

**Table 8:** Types of data classes.

№	Classes	Examples	Object	Definition of the Image
1	ati	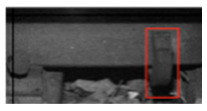	Anti-theft	Not defective
2	bolt_M24	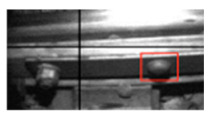	Bolt of the M24 brand	Not defective
3	bolt_M22	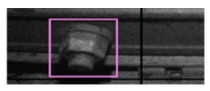	Bolt of the M22 brand	Not defective
4	GBR	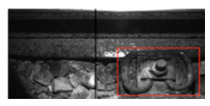	GBR brand sleeper pad	Not defective
5	kpp	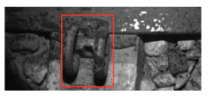	KPP brand sleeper pad	Not defective
6	SKL	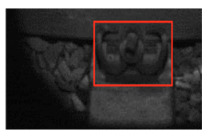	SKL brand sleeper pad	Not defective
7	D65	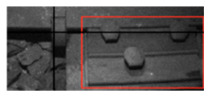	D65 brand sleeper pad	Not defective
8	KD65	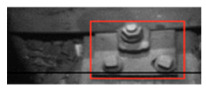	KD65 brand sleeper pad	Not defective
9	D65no16X16	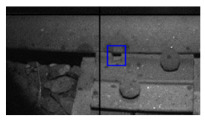	Missing crutch brand 16 × 16	Defective
10	KB65	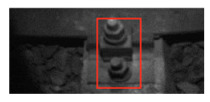	KB65 brand sleeper pad	Not defective
11	IzoStyk	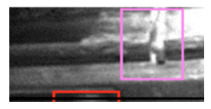	Insulating joint	Not defective
12	KB65NB	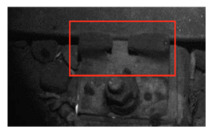	Missing bolt on the KD65 brand sleeper lining	Defective
13	no_M22	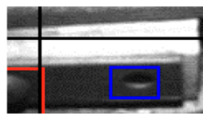	No bolt	Defective
14	NKPPO1	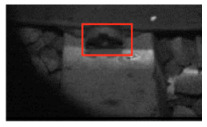	No KPP brand lining	Defective
15	no_SKL	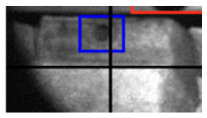	No SKL brand lining	Defective
16	BCW	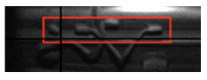	Defective connecting wire	Not defective
17	stuck_styk	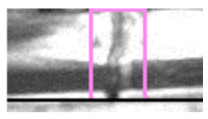	Stuck-together joint	Defective
18	CWWI	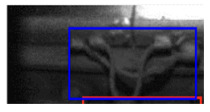	Connecting wire	Not defective
19	BSO1	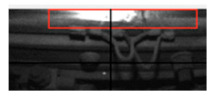	Defective connecting joint	Defective

## Data Availability

All data relevant to the research are provided at https://drive.google.com/drive/folders/1YkLBCdRg_wzf4EVgmiI87qzj02AwH6g4?usp=sharing (accessed on 25 January 2024).
